# Small molecule Z363 co‐regulates TAF10 and MYC via the E3 ligase TRIP12 to suppress tumour growth

**DOI:** 10.1002/ctm2.1153

**Published:** 2023-01-13

**Authors:** Yan Xiong, Lulu Wang, Shiyao Xu, Beibei Fu, Yuchen Che, Mohamed Y. Zaky, Rong Tian, Rui Yao, Dong Guo, Zhou Sha, Feng Lin, Xiaoyuan Lin, Haibo Wu

**Affiliations:** ^1^ Department of Physiology, School of Life Sciences Chongqing University Chongqing China; ^2^ Molecular Physiology Division Zoology Department Faculty of Science Beni‐Suef University Beni‐Suef Egypt; ^3^ Department of Oncology Faculty of Medicine Linköping University Sweden; ^4^ Department of Biomedical and Clinical Sciences Faculty of Medicine Linköping University Sweden; ^5^ Department of Pathology Chongqing Hygeia Hospital Chongqing China

**Keywords:** co‐regulation, phosphorylation, TAF10, TRIP12, ubiquitination

## Abstract

**Background:**

The MYC oncoprotein, also known as the master regulator of genes, is a transcription factor that regulates numerous physiological processes, including cell cycle control, apoptosis, protein synthesis and cell adhesion, among others. MYC is overexpressed in approximately 70% of human cancers. Given its pervasive role in cancer biology, MYC down‐regulation has become an attractive cancer treatment strategy.

**Methods:**

The CRISPR/Cas9 method was used to produce KO cell models. Western blot was used to analyzed the expressions of MYC and TATA‐binding proteinassociated factors 10 (TAF10) in cancer cells (MCF7, A549, HepG2 cells) Cell culture studies were performed to determine the mechanisms by which small molecules (Z363119456, Z363) affects MYC and TAF10 expressions and functions. Mouse studies were carried out to investigate the impact of Z363 regulation on tumor growth.

**Results:**

Z363 activate Thyroid hormone Receptor‐interacting Protein 12 (TRIP12), which phosphorylates MYC at Thr58, resulting in MYC ubiquitination and degradation and thereby regulating MYC target genes. Importantly, TRIP12 also induces TAF10 degradation, which reduces MYC protein levels. TRIP12, an E3 ligase, controls MYC levels both directly and indirectly by inhibiting MYC or TAF10 activity.

**Conclusions:**

In summary,these results demonstrate the anti‐cancer properties of Z363, a small molecule that is co‐regulated by TAF10 and MYC.

## Graphical abstract

Z363 exerts an anti‐cancer effect on cancer cell and xenograft models by co‐regulating TAF10 and MYC. TRIP12 plays an important role in Z363‐induced cancer cell apoptosis by directly and indirectly inhibiting MYC activity. Z363 promotes MYC ubiquitination by activating its phosphorylation. Z363 induces TAF10 degradation, which reduces MYC protein levels.

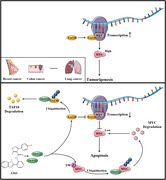

## 1 INTRODUCTION

MYC is a member of the same family as MYCL (L‐Myc) and MYCN (N‐Myc).^[^ ^1^, ^2^, ^3^ ^]^ N‐Myc expression is tissue restricted, and N‐Myc could substitute for MYC in murine development.^[^ ^4^ ^]^ In contrast, the role of L‐Myc is less well understood.^[^ ^4^ ^]^ MYC is located at the intersection of multiple growth‐promoting signal transduction pathways and is an immediate early response gene downstream of numerous ligand–membrane receptor complexes.^[^ ^5^, ^6^ ^]^ MYC expression is tightly controlled by several mechanisms involving numerous transcriptional regulatory motifs found in its proximal promoter region.^[^ ^7^, ^8^, ^9^ ^]^ The MYC proto‐oncogene itself, as well as the mRNA and MYC protein, are tightly regulated by transcription.^[^ ^10^, ^11^ ^]^ MYC is regulated by numerous transcription factors, including CNBP, FBP and TCF.^[^ ^1^, ^9^ ^]^ BRD4, a transcriptional regulator containing a BET domain, was recently shown to bind to the MYC promoter region and play a crucial role in MYC expression in human cancer cells, such that a BET domain chemical inhibitor is able to inhibit tumorigenesis in vivo.^[^ ^12^ ^]^

TAF10 regulates transcription in numerous types of cancer cells.^[^ ^13^, ^14^ ^]^

TAF10, as a chromatin and transcriptional regulator, plays an essential role in numerous cellular processes, including transcription, the cell cycle and apoptosis.^[^ ^15^ ^]^ In mouse F9 embryonal carcinoma cells, absence of TAF10 has been reported to result in cell cycle arrest and apoptosis.^[^ ^14^, ^16^ ^]^ We hypothesised that TAF10 also enhances the transcriptional activity of MYC, resulting in a higher level of MYC protein expression.

MYC appears to be at the intersection of numerous important biological pathways and processes involved in the growth and proliferation of neoplastic cells.^[^ ^17^, ^18^, ^19^, ^20^ ^]^ MYC is implicated in various cancers, with its expression estimated to be elevated or dysregulated in up to 70% of human cancers.^[^ ^1^ ^]^ There are now methods for inhibiting MYC expression, preventing MYC‐MAX dimerisation, inhibiting MYC‐MAX DNA binding and interfering with key MYC target genes.^[^ ^21^ ^]^ Recent strategies have directly targeted MYC, such as BRD4 inhibitors.^[^ ^12^ ^]^ Several pioneering studies have demonstrated that small molecules can directly inhibit MYC activity,^[^ ^21^, ^22^, ^23^ ^]^ but they still lack clinical application. Therefore, the need for chemical probes that directly modulate MYC function and that can serve as possible therapeutic leads remains acute.

## 2 MATERIALS AND METHODS

### 2.1 Chemistry

All small molecules were purchased from Chemieliva Pharmaceutical Co., Ltd. (Chongqing, China). And all small molecules were dissolved in DMSO (Sigma; D2650). First, the small molecules were evaluated for inhibitory activity against cancer lines to validate the availability of anti‐cancer by CCK‐8 Kit assay. Encouragingly, one of the 10 small molecules exhibited extensively anti‐cancer activity (Table S1).

### 2.2 Human specimens

The Chongqing Cancer Hospital obtained breast, lung and liver tumour samples (∼1–4 cm^3^) through re‐sections. Samples were handled and processed in accordance with HTA guidelines. During tumour tissue re‐sectioning at the Chongqing University Cancer Hospital, adjacent non‐tumorous tissue (∼1 cm^3^) was extracted. Every patient gave informed consent. Samples were collected and confirmed to be tumorous or normal. The study was conducted with Institutional Review Board approval.

### 2.3 Mice and ethics statement

Balb/c female nudist mice (6–8 weeks old) were purchased from Hunan SJA Laboratory Animal Co., Ltd. (Hunan, China). The cells (WT cells, MYC KO cells, TAF10 KO cells, DKO cells, WT cells) were injected via SC route in the right hind flank, in 100 μl of phosphate‐buffered saline (PBS), into five groups of BALB/c mice (*n* = 5 each). Then, we observed the tumour development for 4 weeks.

Mice received a single dose of Z363 (60 mg/kg, twice daily) and delivered via i.h. injection for 2 weeks. After the final Z363 dose, all mice were sacrificed, and tumours were collected and weighed. The tumour volume was calculated using a ruler and the following formula: *V* = 0.5 × *L* × *W* ^2^, where *L* represents tumour length and *W* to width.

All animal experimental procedures complied with institutional regulations in accordance with the Animal Welfare Guidelines of Chongqing University.

### 2.4 Cell culture and transfection

MCF7, A549, HepG2 cells (HTB‐22, CCL‐185, HB‐8065) were obtained from the American Type Culture Collection (ATCC, Manassas, VA). Cells were cultured in DMEM medium (Gibco, San Jose, CA, USA) contained 10% (v/v) foetal bovine serum (Gibco). All cells were kept in humidified incubator at 37°C with CO_2_.

Subsequently, tissue samples were mechanically dissociated using a scalpel and transferred to a solution of 20 mg/ml collagenase I (Gibco BRL Co, Invitrogen) in a DMEM medium and incubated at 37°C for 15 h. Dissociated specimens were washed with 10 excess PBS, and separated cells were collected by centrifugation at 300×*g*. Cells were cultured in DMEM supplemented with 10% FBS.

Cells were grown to 70% confluence before transfection. Cells were transfected (Lipofectamine 3000 reagent; Thermo Fisher Scientific) based on the manufacturer's protocols.

### 2.5 Plasmid construction

MYC (NCBI accession number: NM001354870.1) and TAF10 (NM006284.4) coding sequences were inserted into pCMV‐Flag and pCMV‐HA vectors, respectively, as indicated. Primers consist of the following: Flag‐MYC, F‐ATATGGATCCTCCTTGCAGCTGCTTAGACGCTG, R‐TATATCTAGACGCACAAGAGTTCCGTAGCTGT; Flag‐TAF10, F‐ATATAAGCTTATGAGCTGCAGCGGCTCCGGCGCGG, R‐ATATGAGCTCGGGTACCTCCTGAACTGGGG; HA‐TAF10, F‐ATATAAGCTTATGAGCTGCAGCGGCTCCGGCGCGG, R‐AATTTCTAGACCATACTCGCTGAGGGCAGGGGTCA. Underlines represent the restriction sites. Ribobio chemically synthesised control siRNA and siRNAs that target TAF10 and TRIP12 (NCBI accession number: NM001284214.2) (Guangzhou, Guangdong, China). The Lipofectamine RNAiMAX reagent (Thermo Fisher Scientific) was used to transfect siRNAs according to the manufacturer's instructions. The siRNA sequences were listed in Table S2. Addgene was used to acquire the wild‐type Ubiquitin and additional mutants.

### 2.6 Cell proliferation assay

The cell proliferation ability was detect using a Mouse Ki67 ELISA Kit (BD Biosciences, Franklin Lakes, NJ, USA) according to the instructions provided by the manufacturer. Briefly, cells were seed in a 96‐well plate and transfected with GFP‐TRIP12 or si‐TRIP12. After 24 h, cells treated with or without Z363 were compared. Cell lysates were harvested to detect cell proliferation.

### 2.7 Colony formation analysis

One thousand cells were transfected with the Control‐vec or HA‐labelled TAF10 for 24 h and then plated in six‐well culture plates and cultured for 10 days. These cells were fixed with 10% formaldehyde and then stained with 0.05% crystal violet solution. Microscopy was used to acquire images (Carl Zeiss, Jena, Germany).

One thousand MCF7 cells were seeded into each well of a six‐well plate. The cells were then transfected with GFP‐TRIP12 or si‐TRIP12, respectively. 24 h later, cells treated with or without Z363 were compared. The steps for crystal violet staining are outlined above.

### 2.8 Cell apoptosis by Caspase 3 activity analysis

The Caspase 3 Activity Assay Kit (Biomol Research Laboratories, Plymouth Meeting, PA, USA) was used to analyse the activity of Caspase 3. According to the instructions, cell lysates were harvested and incubated overnight at 37°C with Ac‐DEVD‐pNA.

Record the absorbance data at 405 nm in VICTOR X5 Multilabel Plate Reader (PerkinElmer, Waltham, MA, USA).

### 2.9 Cell apoptosis by flow cytometry analysis

An Annexin V‐FITC and propidium iodide (PI) Kit (BD Biosciences) was used to analyse the cell apoptosis. According to the instructions, cells were collected and labelled with PI and Annexin V. Finally, a flow cytometer was used for analysis.

### 2.10 RNA extraction

According to the manufacturer's instructions, total RNA was extracted with Trizol (Thermo Fisher Scientific) reagent. Quantitative PCR experiments were performed using a SuperScript III One‐Step RT‐PCR kit (Thermo Fisher Scientific). Briefly, this procedure included 30 s of pre‐incubation at 95°C and 40 cycles of denaturation at 95°C for 5 s and annealing for 30 s at 60°C. The data were expressed using 2^−ΔΔCt^ method.

The following primers were designed: MYC‐F: 5′‐CCCTAGTGCTGCATGAGGA‐3′, MYC‐R: 5′‐CCTCTTCTCCACAGACACCA‐3′; TAF10‐F: 5′‐GAAGTGAAGCCCGTAGTGTCC‐3′, TAF10‐R: 5′‐ATTGATGCCATACTCGCTGAG‐3′; GAPDH‐F: 5′‐CTGGGCTACACTGAGCACC‐3′, GAPDH‐R: 5′‐AAGTGGTCGTTGAGGGCAATG‐3′.

### 2.11 Immunoblot and immunoprecipitation

Total proteins were extracted and performed the immunoblot analyses. Primary antibodies used: MYC (1:1000; NOVUS; NB600‐336SS), pT58‐MYC (1:1000; Abcam; ab28842), pS62‐MYC (1:1000; Abcam; ab78318), TAF10 (1:800; NOVUS; NBP1‐80706), TRIP12 (1:1000; NOVUS; NBP2‐94325), PCNA (1:1000; Thermo Fisher Scientific; MA5‐11358), Ki67 (1:500; Santa Cruz; sc‐23900), Cleaved‐Caspase 3 (1:500; Abcam; ab2302), Cleaced‐PARP1 (1:1000; Abcam; ab4830), p53 (1:500; Thermo Fisher Scientific; MA5‐12557), Flag (1:1000; Beyotime; AF5051), HA (1:1000; Beyotime; AF5057), GFP (1:1000; Beyotime; AG281) and GAPDH (1:1000; Thermo Fisher Scientific; PA1‐987). Lysates were incubated overnight at 4°C with the appropriate antibodies and Protein A/G beads (Thermo Fisher Scientific) for Co‐IP, followed by immunoblots.

### 2.12 Luciferase assays

The relative activity of MYC promoter was determined by Dual‐Luciferase reporter assay as previously described.^[^ ^52^ ^]^ MYC promoter was amplified from genomic DNA of 293T cells by polymerase chain reaction (PCR) and cloned into luciferase reporter construct pGL4.10 (Promega) and confirmed by sequencing. Considering that TAF10 is a cofactor of the transcription factor IID (TFIID) complexes, which recognise the conserved TATA‐box to position the polymerase properly, the native TATA box sequence TATATAAA was mutated into GGTCTGC on the MYC promoter by overlap extension PCR and defined as pGL4.10‐MYC‐MUT. MCF7, A549 and HepG2 cells were seeded in 24‐well plates and transfected with pGL4.10‐MYC or pGL4.10‐MYC‐MUT plasmids by VigoFect (Vigorous Biotech, Beijing). Luciferase activities were measured 24 h after transfection using the Dual‐Luciferase reporter assay system (Promega). The pRL‐SV40 renilla luciferase reporter (Promega) was included in all transfections for normalisation.

### 2.13 Generation of CRISPR‐Cas9‐based knockout cells

Next, using the CRISPR‐Cas9 method, MYC KO, TAF10 KO and DKO cells were produced. sgRNAs were designed (MYC KO‐sgRNA: CTTCGGGGAGACAACGACGG; TAF10 KO‐sgRNA: GGTTTACGTACTGCCGAGCG) and ligated into the pSpCas9 (BB)‐2A‐Puro (PX459) plasmid following Bbs I digestion. The recombinant was then transfected into MCF7 cells, A549 or HepG2 cells by using Lipo 3000 Transfection Reagent (Invitrogen). Forty‐eight hours after transfection, puromycin (3 μg/ml) was used for the screening of individual colonies. The remaining cells were used for limiting dilution to obtain the cell clone after half of the cells were removed for Western blotting analysis.

### 2.14 Statistical analysis

Statistical analysis was done using GraphPad Prism 5 software. Data are presented as mean ± standard error of the mean (SEM). The difference between the control and the experimental groups was analysed using ANOVA and *t*‐test. A *p* value of ≤.05 was considered statistically significant.

## 3 RESULTS

### 3.1 TAF10 enhances MYC transcriptional activity

**FIGURE 1:**
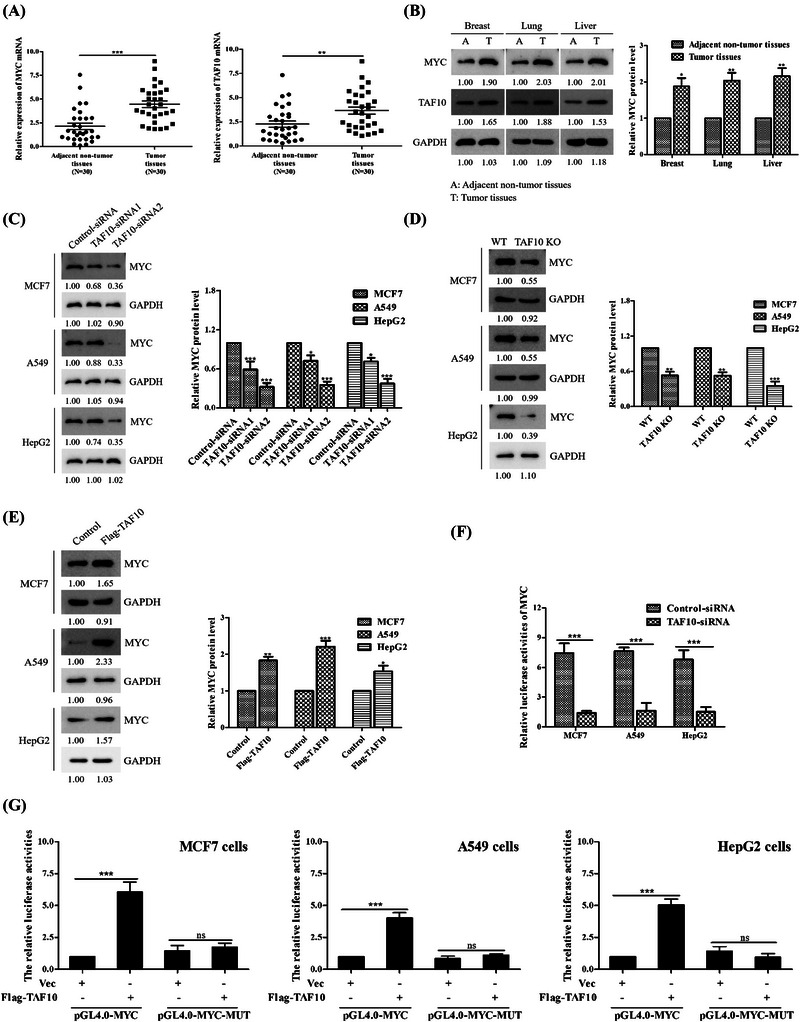
TAF10 enhances MYC transcriptional activity. (A) Real‐time quantitative PCR was used to determine the expression level of TAF10 and MYC in breast, lung, liver cancer tissues and corresponding adjacent non‐tumour tissues in 30 patients. (B) Tumour and adjacent non‐tumour tissues were obtained for primary culture. The levels of MYC and TAF10 proteins were evaluated using Western blotting. (C) Immunoblotting demonstrates the specific knockdown effect of TAF10 siRNA on endogenous MYC protein. TAF10 siRNA and Control siRNA plasmids were transfected into MCF7, A549 and HepG2 cells, respectively. Anti‐MYC or GAPDH antibody was used to probe whole‐cell extracts that were prepared 24 h after transfection. (D) Quantification of MYC expression by Western blot in TAF10 KO cells. Level of relative MYC expression after TAF10 knockout in MCF7, A549 and HepG2 cells. (E) Overexpression of TAF10 determined the MYC protein concentration. MCF7 cells were transfected with Flag‐labelled TAF10, and the endogenous MYC protein level was determined by Western blotting. (F) Luciferase reporter assays in control and TAF10 knockdown cells. MCF7, A549 and HepG2 cells were respectively transfected with Control siRNA or TAF10 siRNA. The MYC promoter activity was analysed using the dual luciferase reporter assay 24 h later. (G) TAF10 is capable of activating MYC‐responsive promoters. MCF7, A549 and HepG2 cells were transfected for 24 h with an empty vector (Vec) or Flag‐labelled TAF10 (Flag‐TAF10). The dual luciferase reporter assay was used to evaluate the activity of the MYC promoter. The *t*‐test was applied to the data in column A. Using a two‐way ANOVA, F's data were analysed. The data displayed in G were analysed using a one‐way ANOVA. The blots represented three independent experiments. All data are presented as the mean ± SEM of *n* = 3. ***p < .001, **p < .01, ns, no significance

First, using breast, lung and liver cancer samples collected from 30 patients at a hospital, we examined the difference in relative mRNA expression of MYC and TAF10 between adjacent non‐tumour tissues and cancer tissues. Our results showed that MYC and TAF10 were significantly more highly expressed in cancer tissues than in adjacent non‐tumour tissues (Figure 1A). MYC was highly expressed in cancer tissues as was TAF10 at the protein level (Figure 1B). TAF10 siRNA (validated in Figure S1A) was transfected into cancer cells expressing endogenous MYC in order to determine whether TAF10 is required for MYC to stimulate transcriptional activity. Figure 1C demonstrates that at 24 h after transfection, TAF10 siRNA effectively inhibited expression of the MYC protein. Next, TAF10 knockout was performed using the CRISPR/Cas9 method based on MCF7, A549, HepG2 cell lines (Figure S1B). As predicted, elimination of endogenous TAF10 decreased MYC protein levels (Figure 1D). As depicted in Figure 1E, TAF10 overexpression (validated in Figure S1C) increased expression of MYC. These results suggest that TAF10 is a crucial transcription factor for MYC. MCF7 cells were transfected with either control siRNA or TAF10 siRNA to examine the role of endogenous TAF10 in boosting MYC transcriptional activity. Suppression of MCF7 cell endogenous TAF10 expression significantly reduced MYC transcriptional activity (Figure 1F). Moreover, overexpression of TAF10 activated wild‐type MYC promoter reporter activity but not mutated MYC promoter reporter activity (Figure 1G). These findings indicate that TAF10 can modulate the transcriptional activity of MYC.

### 3.2 TAF10 promotes cancer cell proliferation and migration

**FIGURE 2:**
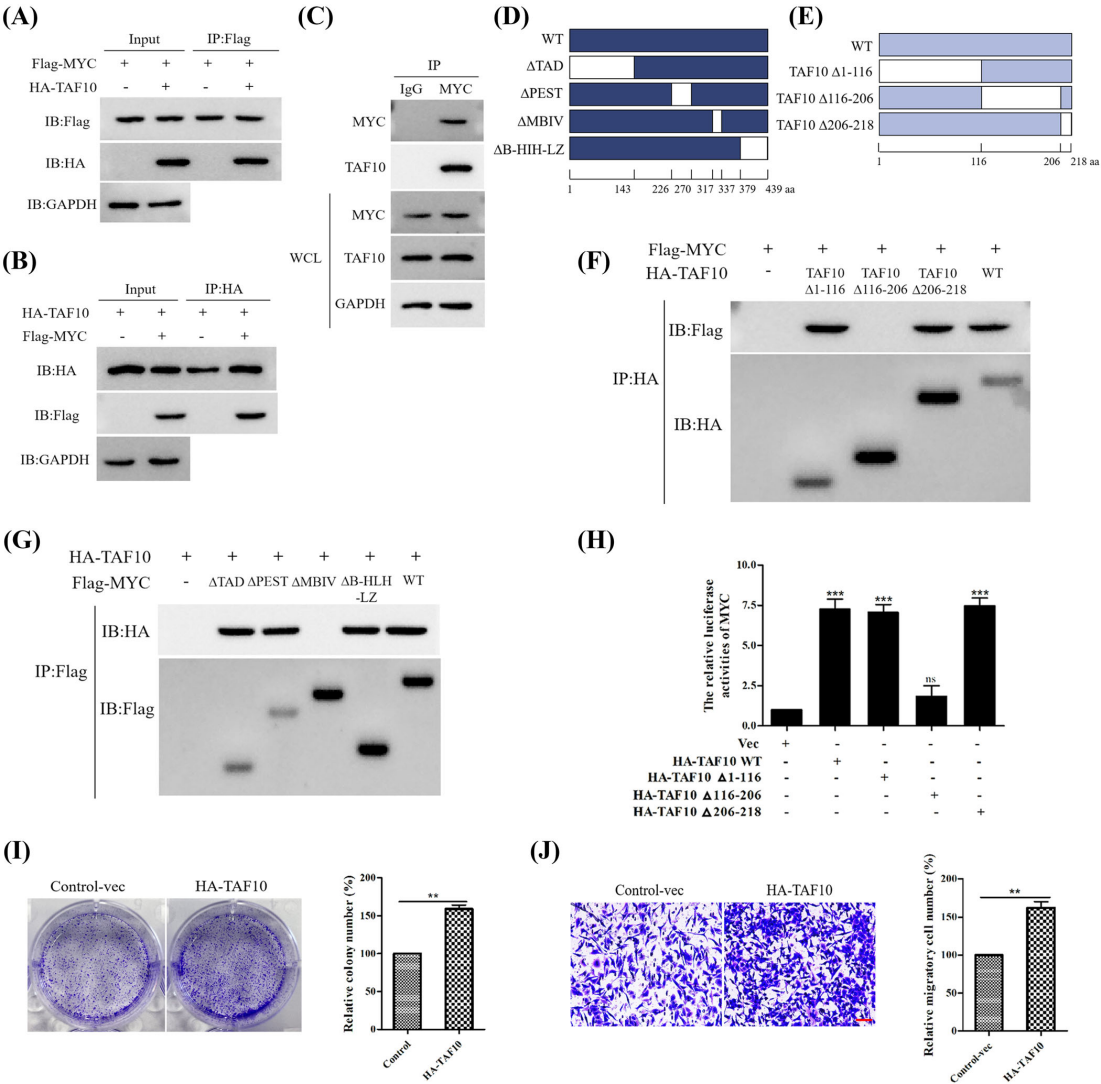
TAF10 promotes cancer cell proliferation and migration. (A and B) MCF7 cells were transfected with Flag‐labelled MYC and HA‐labelled TAF10, and the interaction between MYC and TAF10 was detected by Co‐IP. (C) Endogenous interaction of MYC and TAF10 was tested in MCF7 cells. (D) Schematic representation of MYC mutants. (E) Schematic representation of TAF10 mutants. Co‐IP was used to detect the interaction between the TAF10 mutants and MYC in MCF7 cells. Co‐IP was used to detect the interaction. (G) Interaction between the MYC mutants and TAF10 in MCF7 cells was detected using Co‐IP. (H) MCF7 cells were co‐transfected with empty vector (Vec) and HA‐labelled TAF10 wild‐type (HA‐TAF10 WT) or mutants (HA‐TAF10 Δ1‐116, HA‐TAF10 Δ116‐206, HA‐TAF10 Δ206‐218). The MYC promoter activity was analysed using the dual luciferase reporter assay 24 h later. (I) Overexpression of TAF10 assessed the capacity for colony formation. MCF7 cells were transfected with either Control‐vec or HA‐TAF10. The ability of cells to form colonies was measured using crystal violet staining. (J) Overexpression of TAF10 measured the migration capacity of cells. MCF7 cells were transfected with either Control‐vec or HA‐TAF10. Transwell assays were used to assess the migration capacity of cells. Scale bar, 20 μm. Data shown in H were analysed by one‐way ANOVA. Data shown in I and J were analysed by *t*‐test. The blots represented three independent experiments. All data are presented as the mean ± SEM of *n* = 3. ***p < .001, **p < .01, ns, no significance

We previously demonstrated that TAF10 increases the transcriptional activity of MYC, and we thus examined the effect of MYC's interaction with TAF10. Flag‐labelled MYC and HA‐labelled TAF10 were transfected into MCF7 cells, and Co‐IP demonstrated that TAF10 could be co‐immunoprecipitated with MYC (Figures 2A and B). Furthermore, we found that endogenous MYC interacted with TAF10 (Figure 2C). Both MYC and TAF10 domain organisations were meticulously mapped (Figures 2D and E). To detect the interaction region between MYC and TAF10, we used recombinant TAF10 mutant vector to transfect into cells. The ability of TAF10 mutants lacking both 1–116 aa (Δ1–116) or 206–218 aa of TAF10 (Δ206–218) to interact with MYC was unaffected, whereas a TAF10 mutant (Δ116–206) interacted with MYC. Further evidence demonstrated that MYC bound to the TAF10 domain between residues 116 and 206 (Figure 2F). The TAD, PEST, MBIV and B‐HLH‐LZ fragments were deleted in MYC truncation mutants. TAF10 bound within MBIV of the MYC domain (Figure 2G). TAF10 and MYC interact directly through TAF10 residues 116 and 206 and the MBIV domain of MYC.

We further investigated the role of this region (residues 116–206) in regulating MYC transcriptional activity. As shown in Figure 2H, a mutant HA‐TAF10 Δ116–206, which was constructed by deleting residues 116–206, which significantly attenuated activation of MYC and indicated that the region from residues 116 to 216 of TAF10 is required for TAF10‐mediated activation of MYC.

Given the correlation between TAF10 expression and the development of breast cancer, we further investigated the biological function of TAF10 in breast cancer. As depicted in Figure 2I, TAF10 overexpression significantly promoted MCF7 cell proliferation. Next, we examined the impact of TAF10 on the migratory capacity of breast cancer cells. Transwell assays demonstrated that TAF10 overexpression in MCF7 cells increased cancer cell migration (Figure 2J). Altogether, these findings demonstrate that TAF10 promotes the proliferation and metastasis of breast cancer cells.

### 3.3 Z363 promotes MYC and TAF10 degradation

**FIGURE 3:**
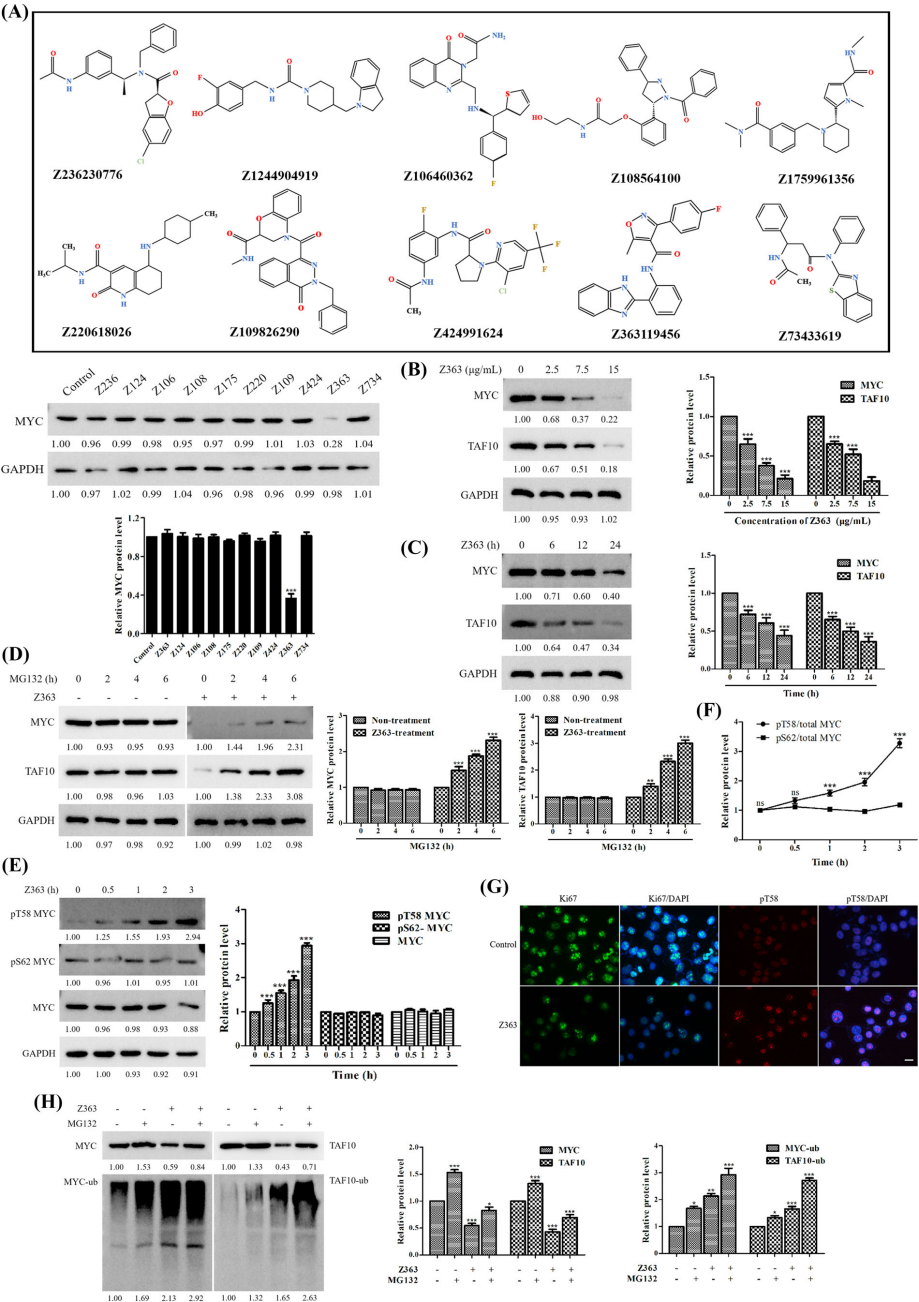
Z363 promotes MYC and TAF10 degradation. (A) Identification of small inhibitory molecules for MYC. (B) MCF7 cells were treated with Z363 (0, 2.5, 7.5 and 15 μg/ml) for 24 h. The protein levels of MYC and TAF10 were analysed by Western blotting. (C) MCF7 cells were treated with Z363 (7.5 μg/ml) for 0, 6, 12 and 24 h. Furthermore, the protein levels of MYC and TAF10 were analysed by Western blotting. (D) MCF7 cells were treated with 25 μM MG132 at the indicated time points, followed by treatment with or without Z363 (7.5 μg/ml) for 24 h, and MYC and TAF10 expressions were analysed by Western blotting. (E) Western blots for MYC, phosphorylated MYC T58 and S62 in MCF7 cells treated with Z363 at the times indicated. (F) Ratios of pT58 or pS62 to total MYC protein levels from the experiment (E). (G) IF staining for Ki67 and pT58 in Z363‐treated MCF7 cells, scale bar, 10 μm. (H) MCF7 cells were treated with 25 μM MG132 for 2 h, followed by Z363 treatment (7.5 μg/ml) for 24 h. Expressions of MYC and TAF10 were assessed using Western blot analysis. Data shown in F were analysed by two‐way ANOVA. Fluorescence images and blots were representative of three independent experiments. All data are presented as the mean ± SEM of *n* = 3. ***p < .001, ns, no significance

Our earlier research demonstrated that 10 small molecules can induce apoptosis in cancer cells (Table S1). In this study, expression levels of MYC were determined by treating MCF7 cells with 10 small molecules. Z363 was discovered to significantly reduce MYC protein levels (Figure 3A). Thus, Z363 can directly target the MYC protein to induce apoptosis in cells. In addition, we discovered that Z363 modulates expression of MYC and TAF10 in a dose‐ (Figure 3B) and time‐dependent manner (Figure 3C). MG132 (proteasome inhibitor) could alleviate the decrease of MYC expression induced by Z363 treatment. This result demonstrated Z363 affects MYC protein stability (Figure 3D). MYC protein stability is regulated by several mechanisms, the most prominent of which is an ordered phosphorylation cascade in which phosphorylation of MYC at serine 62 (pS62) by kinases such as ERK, CDK and JNK primes MYC for phosphorylation at threonine 58 (pT58) by GSK3β.^[^ ^24^, ^25^, ^26^, ^27^ ^]^ E3 ubiquitin ligases recognise MYC pT58, which is then degraded by the 26S proteasome.^[^ ^25^ ^]^ We studied whether the stability of MYC protein was destroyed by Z363 through this mechanism. The expression of MYC pT58 increased (Figures 3E and G), while that MYC pS62 did not significantly difference by Z363 treatment (Figure 3E). More importantly, this increase in pT58 occurred prior to the decrease in MYC protein levels (Figure 3F). In addition, the proliferation marker Ki67 was reduced in tumour tissues following Z363 treatment (Figure 3G). Therefore, we speculate that MYC undergoes ubiquitination degradation.

We further investigated ubiquitination of MYC and TAF10. After Z363 treatment, proteasomal degradation led to a decrease in the MYC protein level (Figure 3H, Lane 3), whereas both the protein and ubiquitination levels of MYC increased by pre‐treating with MG132 (Figure 3H, Lane 4). Furthermore, TAF10 protein and ubiquitination was accumulated by pre‐treating with MG132 (Figure 3H, Lane 8). Thus, our findings suggest that Z363 promotes MYC and TAF10 ubiquitination degradation.

In summary, these results indicate that Z363 promotes MYC and TAF10 degradation. Notably, MYC degradation enhanced MYC phosphorylation on T58.

### 3.4 Z363‐regulated E3 ligases target MYC and stabilise TAF10

**FIGURE 4:**
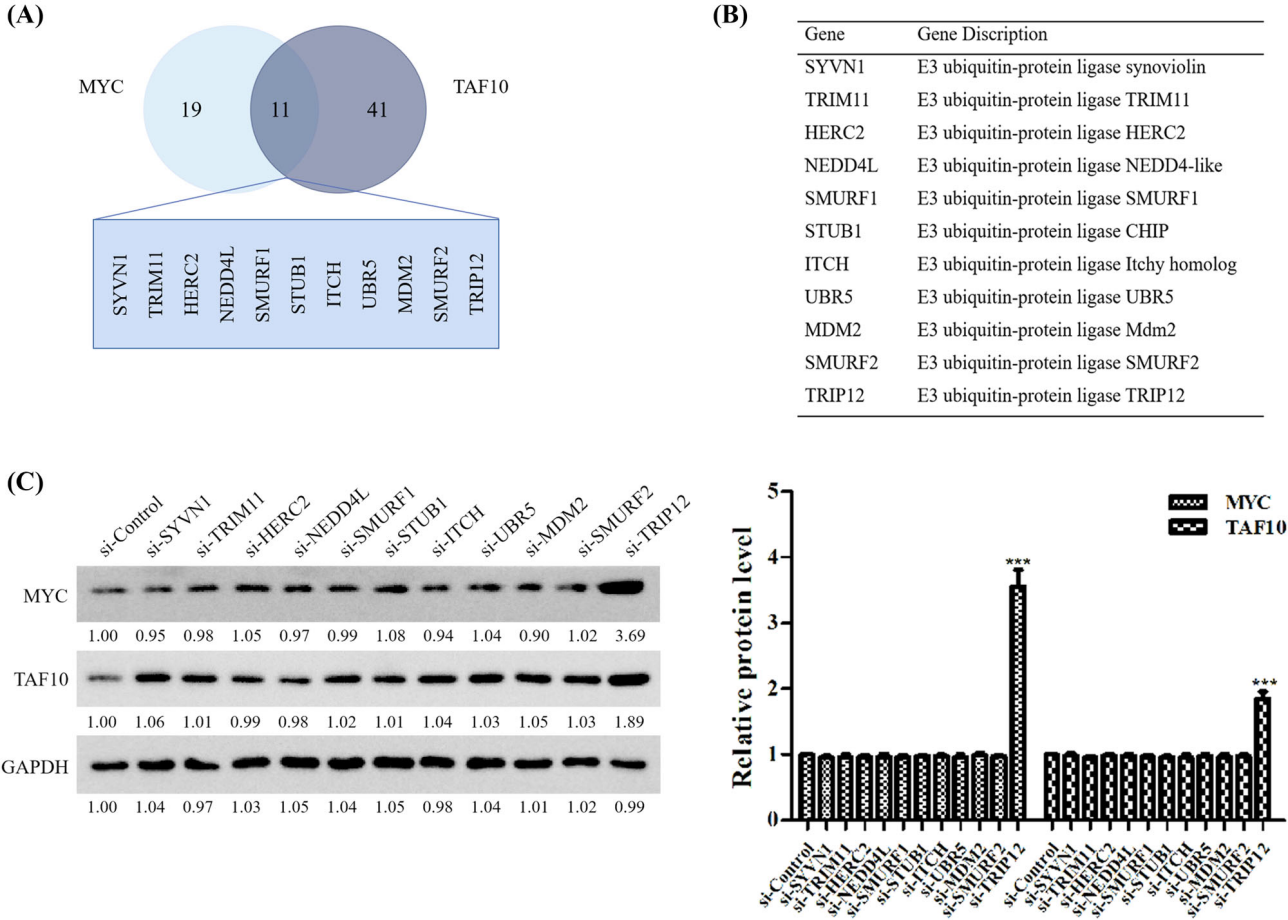
Z363‐regulated E3 ligases target MYC and TAF10 stability. (A) UbiBrowser was utilised to directly predict potential E3 ligases targeting MYC and TAF10. (B) A table displaying eleven E3 ligases that may have targeted MYC and TAF10. (C) siRNA was transfected into MCF7 cells. The expressions of MYC and TAF10 were analysed by Western blotting 24 h later. The blots represented three independent experiments.

As Z363 promotes MYC and TAF10 degradation, this process may be regulated by the manipulation factors of proteasome degradation. Therefore, we used the online tool UbiBrowser (ncpsb.org.cn) to predict potential E3 ligases that directly target MYC and TAF10 (Figure 4A).

According to the prediction results, 19 E3 ligases target MYC and 41 target TAF10, 11 of which are target both MYC and TAF10 (Figure 4B). In our study, we discovered that when TRIP12 was inhibited by siRNA, protein levels of MYC and TAF10 increased significantly (Figure 4C). These results suggest that MYC and TAF10 undergo ubiquitination modification by binding cooperatively to TRIP12 in the presence of Z363.

### 3.5 TRIP12 inhibits breast cancer cell proliferation and metastasis and induces apoptosis

**FIGURE 5:**
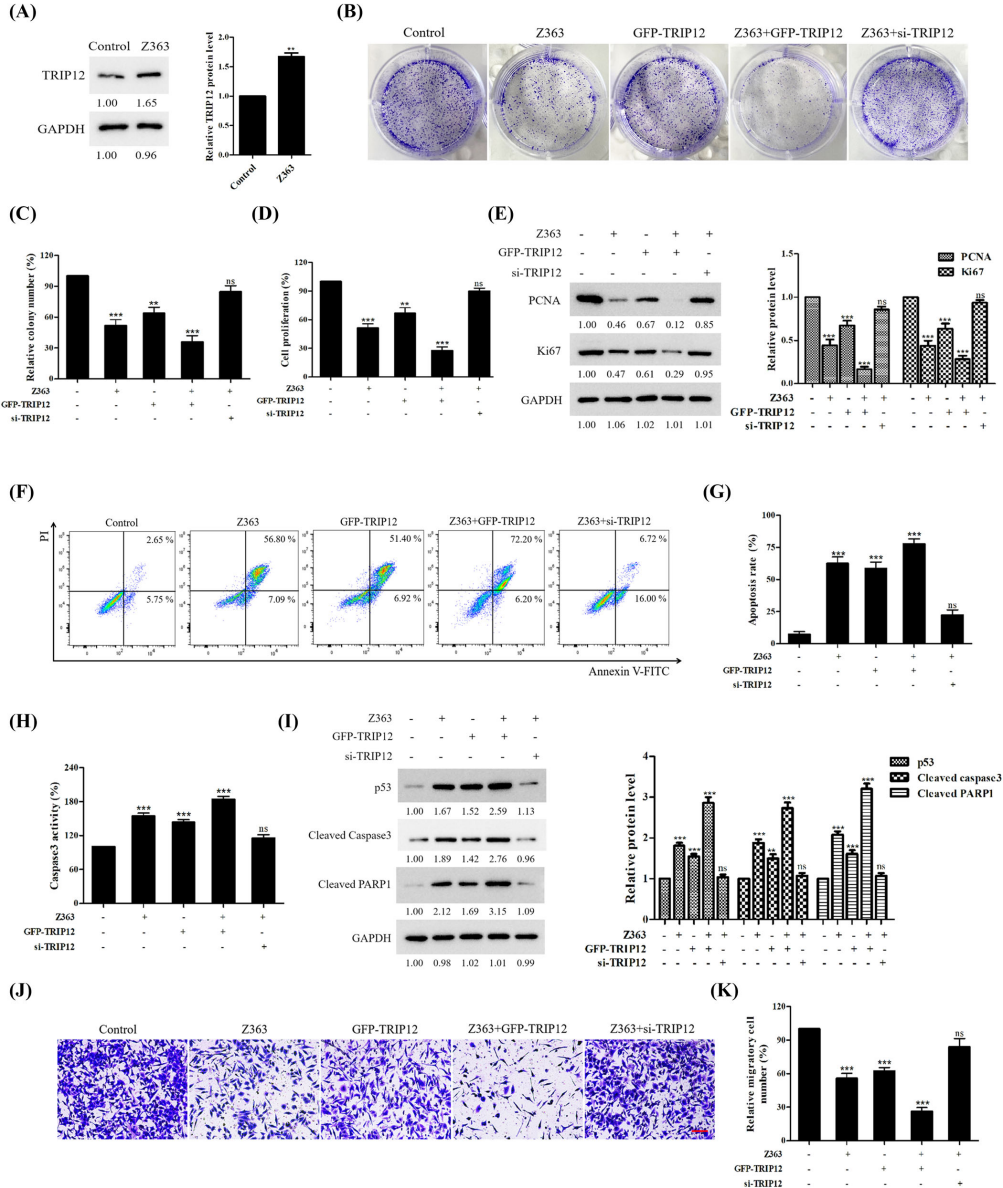
TRIP12 inhibits breast cancer cells proliferation, metastasis and induces apoptosis. (A) MCF7 cells were treated with Z363 (7.5 μg/ml) for 24 h. The protein levels of TRIP12 were analysed by Western blotting. (B) MCF7 cells were transfected with GFP‐TRIP12 or si‐TRIP12 for 24 h, followed by treated with or without Z363 (7.5 μg/ml) for 24 h. The ability of cell to form colonies was measured using crystal violet staining. (C) Statistical analysis of colony formation. (D) MCF7 cells were transfected with GFP‐TRIP12 or si‐TRIP12 for 24 h, followed by treated with or without Z363 (7.5 μg/ml) for 24 h. Cell proliferation of groups were measured by a Ki67 ELISA kit. (E) The expressions of Ki67 and PCNA were analysed by Western blotting. (F) MCF7 cells were transfected with GFP‐TRIP12 or si‐TRIP12 for 24 h, followed by treated with or without Z363 (7.5 μg/ml) for 24 h. MCF7 cells were treated with different groups, followed by assessment of apoptosis by flow cytometry analysis. (G) Statistical analysis of cell apoptosis. (H) Caspase‐3 activity was measured in MCF7 cells treated with different groups. (I) The expressions of Cyto C and p53 were analysed by Western blotting. (J) Cells were transfected with GFP‐TRIP12 or si‐TRIP12 for 24 h, followed by treated with or without Z363 (7.5 μg/ml) for 24 h. Cell migration ability of groups were measured by transwell assays. Scale bar, 20 μm. (K) Statistical analysis of migration ability. Data shown in C, D and G, H, K were analysed by one‐way ANOVA. Colony images, fluorescence images, transwell and blots were representative of three independent experiments. All data are presented as the mean ± SEM of *n* = 3. ***p < .001, **p < .01, ns, no significance

Given the association between TRIP12 expression and tumorigenesis, we investigated the biological function of TRIP12 in breast cancer. Figure 5A demonstrates that Z363 activated TRIP12 in MCF7 cells. As illustrated in Figures 5B and C, both GFP‐TRIP12 overexpression and Z363 significantly inhibited proliferation of MCF7 cells, as measured by colony formation assays. After transfecting cells with GFP‐TRIP12, they were treated with Z363 (7.5 μg/ml) for 24 h, and we observed more severe suppression of cell proliferation (Figure 5B, picture 4). In contrast, this phenomenon was eliminated when cells were transfected with si‐TRIP12 and then treated with Z363 (Figure 5B, picture 5). Ki67 ELISAs also showed similar outcomes in the indicated cells (Figure 5D). Ki67 and proliferating cell nuclear antigen (PCNA) proteins are commonly used to evaluate the growth fraction of a cell population as standard markers of proliferation.^[^ ^28^ ^]^ As shown in Figure 5E, both GFP‐TRIP12 overexpression and Z363 significantly inhibited proliferation of MCF7 cells. We evaluated the impact of TRIP12 on the apoptosis capabilities of breast cancer cells by flow cytometry and a caspase‐3 activity assay. Both GFP‐TRIP12 overexpression and Z363 significantly promoted apoptosis; however, siRNA‐mediated knockdown of TRIP12 alleviated this effect of Z363 on the cells (Figures 5F–H). Then, apoptosis‐related proteins (Cleaved‐PARP1, Cleaved‐Caspase 3) were detected, and Cleaved‐PARP1 and Cleaved‐Caspase 3 expression in MCF7 cells treated with Z363 or transfected with GFP‐TRIP12 was used to detect apoptosis. Western blotting revealed significantly up‐regulated expression levels of Cleaved‐Caspase3 and Cleaved‐PARP1 in MCF7 cells treated with Z363 or transfected with GFP‐TRIP12. More importantly, after transfection with siTRIP12 and treatment with Z363, expression levels of cleaved Caspase3 and cleaved PARP1 in MCF7 cells were not significantly different compared with the control (Figure 5I). p53, as a tumour suppressor, is also involved in apoptosis,^[^ ^29^ ^]^ and the expression level of p53 also showed that TRIP12 knockdown alleviated the apoptosis induced by Z363 (Figure 5I).

Furthermore, we examined the effect of TRIP12 on the migration capacity of breast cancer cells. Transwell assays revealed that both GFP‐TRIP12 overexpression and Z363 significantly inhibited MCF‐7 cell migration but that siRNA‐mediated knockdown of TRIP12 alleviated the inhibitory effect of Z363 on migration (Figures 5J and K).

These findings indicate that TRIP12 performs the same function as Z363 in inhibiting breast cancer cell proliferation and metastasis, along with promoting cancer apoptosis.

### 3.6 TRIP12 decreases MYC protein stability by modulating MYC‐threonine 58 phosphorylation

As shown in Figure 3, Z363 promoted degradation by promoting MYC T58 phosphorylation. As depicted in Figure 4, TRIP12 exerted the same function as Z363. Thus, we hypothesised that overexpression of TRIP12 reduces MYC protein stability by modulating phosphorylation of MYC‐threonine 58.

**FIGURE 6:**
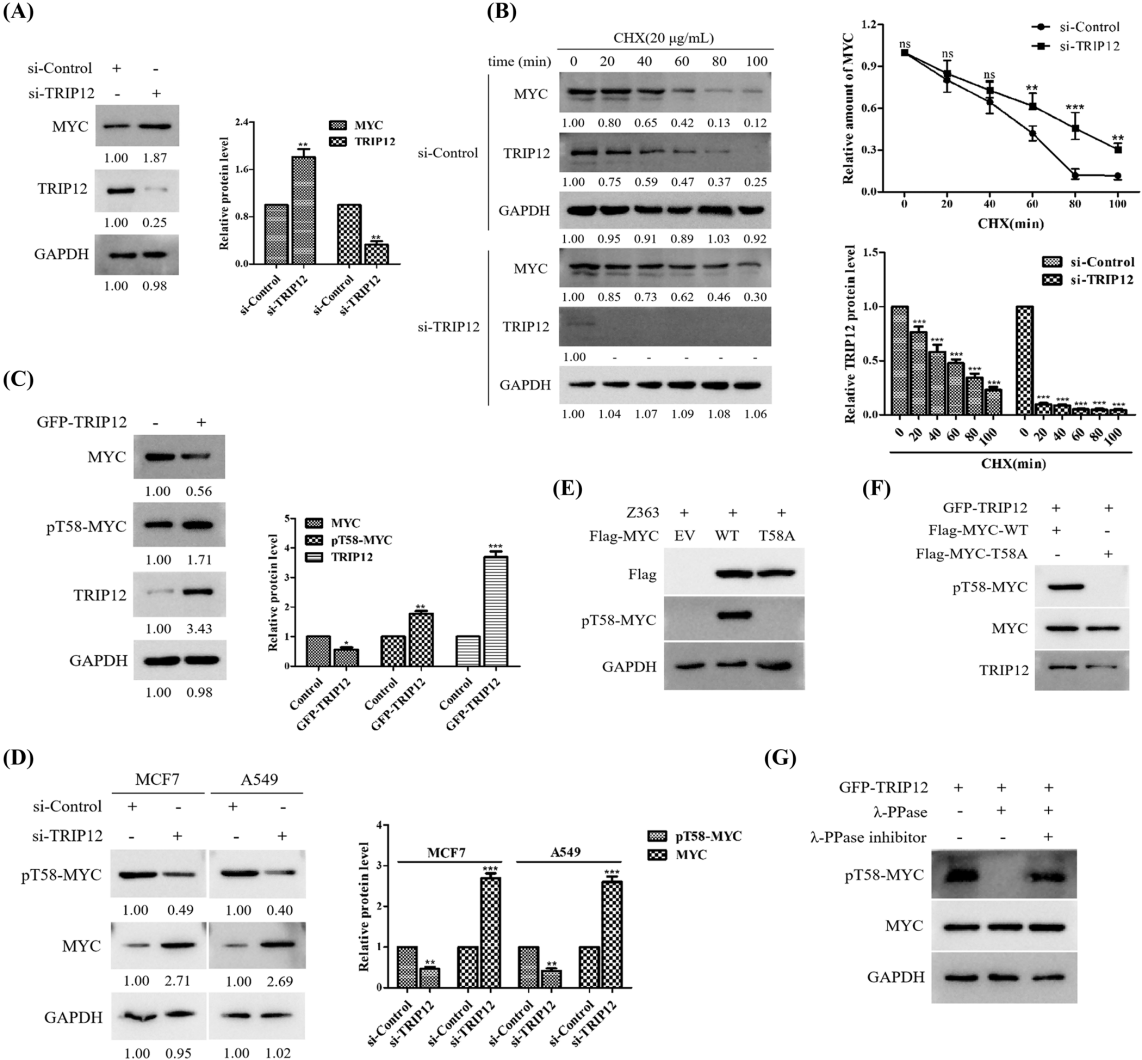
TRIP12 decreases MYC protein stability by modulating MYC‐threonine 58 phosphorylation. (A) MCF7 cells were transfected with Control siRNA or TRIP12 siRNA for 24 h. MYC protein levels were determined by Western blotting. (B) Left panel: MCF7 cells transfected with indicated siRNA for 24 h were treated with CHX (20 μg/ml) for the indicated times and then analysed by Western blotting using indicated antibodies. Right panel: Quantification of the MYC band intensities over time. (C). Overexpression of TRIP12 measured MYC protein level. MCF7 cells were transfected with GFP‐TRIP12, followed by Western blotting with indicated antibodies. (D) MCF7 and A549 cells were transfected with Control or TRIP12 siRNA for 24 h. The expressions of MYC and phosphorylated MYC T58 were analysed by Western blotting. (E) MCF7 cells stably expressing empty vector (EV), wild‐type MYC or MYC T58A mutant were lysed and then analysed by Western blotting using indicated antibodies. (F) Flag‐MYC‐WT or T58A protein were incubated in vitro with immunoprecipitates isolated from MCF7 cells transfected with GFP‐TRIP12 construct and then analysed by Western blotting using indicated antibodies. (G) Lysates prepared from MCF7 cells transfected with GFP‐TRIP12 were treated with λ‐PPase with or without λ‐PPase inhibitor and then analysed by Western blotting as indicated. Data shown in B were analysed by two‐way ANOVA. Blots were representative of three independent experiments. All data are presented as the mean ± SEM of *n* = 3. ***p < .001, **p < .01, ns, no significance

Targeting TRIP12 with siRNAs resulted in MYC accumulation in MCF7 cells, confirming these hypotheses in cells (Figure 6A). In addition, the half‐life of MYC was significantly prolonged after TRIP12 depletion (Figure 6B). These findings indicate that TRIP12 inhibits MYC stability. Comparison of pT58 levels in MCF7 cells transfected with either TRIP12 or a control vector. Notably, endogenous MYC pT58 levels were dramatically increased in cells overexpressing GFP‐TRIP12 (Figure 6C). Conversely, siRNA‐mediated knockdown of TRIP12 decreased endogenous MYC pT58 levels (Figure 6D).

In fact, wild‐type MYC was recognised by the anti‐pT58‐MYC antibody, while MYC T58A mutant (the serine residue at position 58 was replaced with alanine) was not recognised (Figures 6E and F). Moreover, it has similar results by pre‐treatment with λ‐PPase (Figure 6G). These results demonstrate that the anti‐pT58‐MYC antibody is highly specific for recognising Thr58 phosphorylated MYC, and MYC is phosphorylated at this residue in vivo.

Altogether, these findings indicate that overexpression of TRIP12 and Z363 decreases MYC protein stability by modulating MYC‐threonine 58 phosphorylation.

### 3.7 TRIP12 phosphorylation of MYC Thr58 leads to MYC ubiquitination and degradation

The above results demonstrate that TRIP12 is essential for the stability of MYC protein. Similarly, phosphorylation of Thr58 by TRIP12 should reduce MYC stability upon overexpression. MYC Thr58 phosphorylation induces its ubiquitination and subsequent degradation. To determine the mechanisms underlying TRIP12‐mediated MYC degradation, we examined TRIP12's ability to interact with MYC.

**FIGURE 7:**
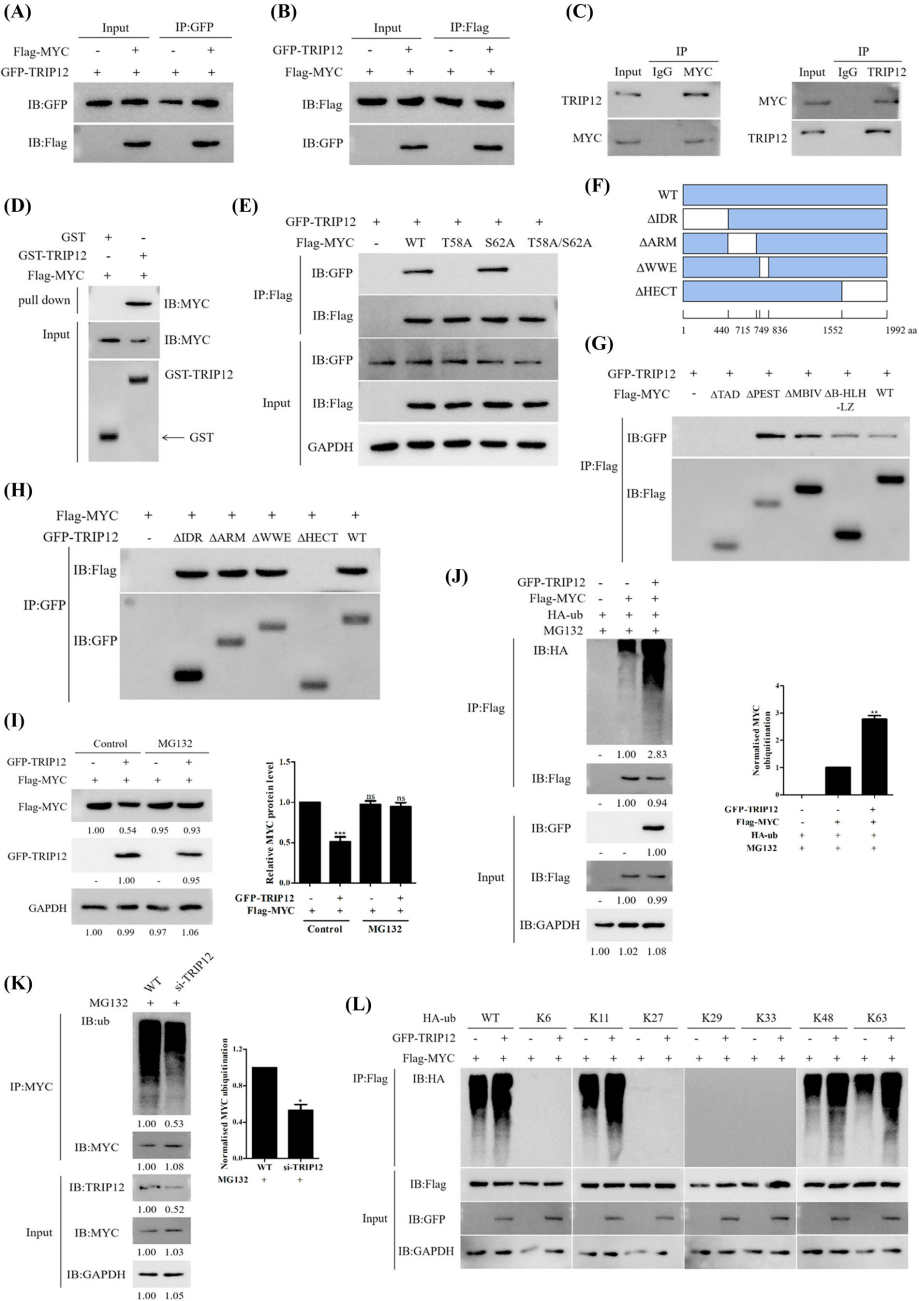
TRIP12 phosphorylation of MYC Thr58 leads to MYC ubiquitination and degradation. (A and B) Flag‐labelled MYC and GFP‐labelled TRIP12 were transfected into MCF7 cells. Cells were treated with Z363 (7.5 μg/ml) for 24 h, and the interaction between MYC and TRIP12 was detected by Co‐IP. (C) Endogenous TRIP12 binds to MYC and vice versa. MCF7 cell lysates were subjected to immunoprecipitation using IgG, anti‐MYC or anti‐TRIP12 antibodies and then analysed by Western blotting as indicated. (D) The interaction between TRIP12 and MYC was detected by GST pulldown assay in vitro. (E) The interaction between the wild‐type MYC as well as its mutants T58A, S62A, T58A/S62A and TRIP12 in MCF7 cells was detected using Co‐IP. (F) Schematic diagram of the TRIP12 mutants. (G) Interaction between the MYC mutants and TRIP12 in MCF7 cells was detected using Co‐IP. (H) Interaction between the TRIP12 mutants and MYC in MCF7 cells was detected using Co‐IP. (I) The proteasome inhibitor MG132 blocks TRIP12‐induced MYC degradation. MCF7 cells were transfected with Flag‐MYC together with either GFP‐TRIP12 or empty vector, MG132 (20 μM) was added to the medium 2 h before protein harvest. (J) HA‐ub, Flag‐MYC, and GFP‐TRIP12 plasmids were transfected alone or co‐transfected into MCF7 cells, and the ubiquitination of MYC was detected by Co‐IP. (K) Ubiquitination of MYC was analysed in WT and si‐TRIP12 MCF7 cells. (L) Lys‐48‐linked ubiquitination catalysed by the wild‐type TRIP12 (GFP‐TRIP12‐WT) was further confirmed by seven Lys‐only ubiquitin mutants, Lys‐6, ‐11, ‐27, ‐29, ‐33, ‐48 and ‐63. Blots were representative of three independent experiments.

Using Co‐IP assay, we examined the interaction between TRIP12 and MYC (Figures 7A and B). Interaction between endogenous TRIP12 and MYC was also confirmed in MCF7 cells (Figure 7C) and by a GST pulldown assay in vitro (Figure 7D). Furthermore, MYC mutant S62A may interact with TRIP12 (Figure 7E).

We further explored the mechanism of TRIP12‐mediated MYC degradation. GFP‐labelled TRIP12 was effectively pulled down by truncated MYC mutants, and GFP‐labelled TRIP12 was pulled down by the TAD domain (Figure 7G). We also explored interaction between MYC and the mutant TRIP12 and found that MYC interacts with the HECT domain of TRIP12 (Figures 7F and H). GFP‐TRIP12 and Flag‐MYC were transfected into cells. Addition of MG132 (proteasomal inhibitor) could effectively blocked TRIP12‐induced MYC degradation (Figure 7I), indicating that TRIP12 mediates proteolytic degradation of MYC. We conducted ubiquitination assays to confirm that TRIP12 catalyses the formation of polyubiquitin chains by MYC (Figure 7J). However, when TRIP12 was inhibited by siRNA, MYC was less ubiquitinated (Figure 7K).

Ubiquitin contains seven lysine residues (Lys‐6, Lys‐11, Lys‐27, Lys‐29, Lys‐33, Lys‐48 and Lys‐63), which can participate in polyubiquitination or uniubiquitination, thus involving in a variety of biological functions.^[^ ^30^, ^31^ ^]^ Polyubiquitination of Lys‐48 link is related to proteasome degradation, while polyubiquitination of Lys‐63 link is related to multiple functions.^[^ ^25^ ^]^ We conducted additional tests to determine whether TRIP12 catalyses Lys‐48‐linked polyubiquitination of MYC. We then performed polyubiquitination assays with seven Lys‐only mutants of ubiquitin (Lys‐6, Lys‐11, Lys‐27, Lys‐29, Lys‐33, Lys‐48 and Lys‐63). These mutants retain only one lysine, with the others being converted to arginine. In the presence of Lys‐48 mutant, TRIP12 catalysed MYC polyubiquitination (Figure 7L). Therefore, TRIP12 is able to catalyse MYC to form Lys‐48‐linked polyubiquitin chains, which is consistent with its role in facilitating MYC degradation by proteasomes.

### 3.8 TRIP12 interacts with TAF10 and inhibits MYC activity

TRIP12, as an E3 ligase, also regulated TAF10 ubiquitination (Figure 3H). Given the diverse functional consequences of TAF10 ubiquitination by TRIP12, we investigated the possibility that TRIP12 and TAF10 interact dynamically to regulate MYC levels.

**FIGURE 8:**
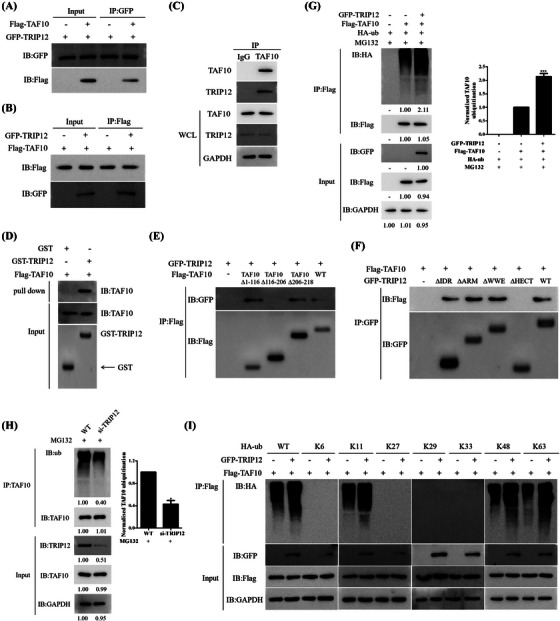
TRIP12 interacts with TAF10 and inhibits MYC activity. (A and B) Flag‐labelled TAF10 and GFP‐labelled TRIP12 were transfected into MCF7 cells. Cells were treated with Z363 (7.5 μg/ml) for 24 h, and the interaction between TAF10 and TRIP12 was detected by Co‐IP. (C) Endogenous TRIP12 binds to TAF10 and vice versa. MCF7 cell lysates were subjected to immunoprecipitation using IgG, anti‐TAF10 or anti‐TRIP12 antibodies and then analysed by Western blotting as indicated. (D) The interaction between TRIP12 and TAF10 was detected by GST pulldown assay in vitro. (E) Interaction between the TAF10 mutants and TRIP12 in MCF7 cells was detected using Co‐IP. (F) Interaction between the TRIP12 mutants and TAF10 in MCF7 cells was detected using Co‐IP. (G) HA‐ub, Flag‐TAF10 and GFP‐TRIP12 plasmids were transfected alone or co‐transfected into MCF7 cells, and the ubiquitination of TAF10 was detected by Co‐IP. (H) Ubiquitination of TAF10 was analysed in WT and siTRIP12 MCF7 cells. (I) Lys‐48‐linked ubiquitination catalysed by the wild‐type TRIP12 (GFP‐TRIP12‐WT) was further confirmed by seven Lys‐only ubiquitin mutants, Lys‐6, ‐11, ‐27, ‐29, ‐33, ‐48 and ‐63. Blots were representative of three independent experiments.

Co‐IP demonstrated a direct interaction between TRIP12 and TAF10 (Figures 8A and B). Indeed, endogenous TRIP12 co‐immunoprecipitated with TAF10 in MCF7 cells (Figure 8C), and *Escherichia coli*‐expressed GST‐TRIP12 pulled down Flag‐TAF10 (Figure 8D). Using TRIP12 truncation and deletion mutants, the interaction domain that binds TAF10 to TRIP12 was mapped. Consequently, deletion of TAF10 116–206 aa significantly inhibited TRIP12 binding (Figure 8E). Accordingly, the TRIP12 mutant lacking the HECT domain (ΔHECT) did not effectively pull down TAF10 (Figure 8F), and the 116–206 aa segment of TAF10 bound to the HECT domain of TRIP12. We conducted ubiquitination assays to confirm that TRIP12 can catalyse TAF10 in forming polyubiquitin chains (Figure 8G). However, when TRIP12 was inhibited by siRNA, TAF10 was less ubiquitinated (Figure 8H). In the presence of Lys‐48 mutant, TRIP12 catalysed TAF10 polyubiquitination (Figure 8I). These observations suggest that TRIP12 catalyses TAF10 to form Lys‐48‐linked polyubiquitin chains, leading to TAF10 degradation, thereby inhibiting MYC activity.

### 3.9 Co‐inhibition of MYC and TAF10 causes a synergistic reduction in cell proliferation and tumour growth

**FIGURE 9:**
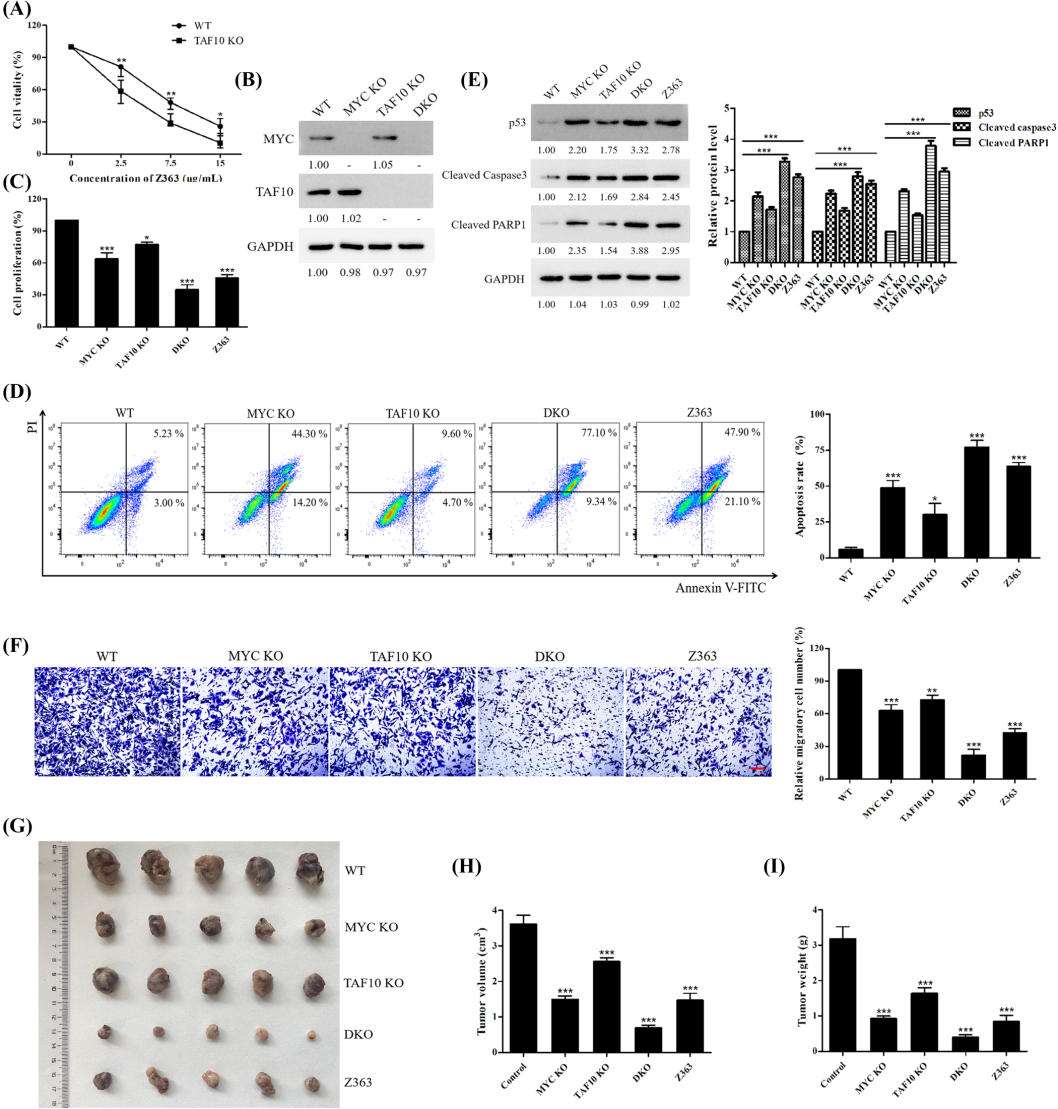
Co‐inhibition of MYC and TAF10 causes synergistic reduction of cell proliferation and tumour growth. (A) Cells (WT, TAF10 KO) were treated with different doses of Z363 for 24 h. Cell proliferation was determined by the CCK‐8 assay. (B) Knockout reliability was detected by Western blotting. (C) The proliferation of wild‐type cells (WT), single and double KO cells (MYC KO, TAF10 KO, DKO) and Z363‐treated cells were detected by Ki67 ELISA kit. (D) The apoptosis of wild‐type cells (WT), single and double KO cells (MYC KO, TAF10 KO, DKO) and Z363‐treated cells were detected by flow cytometry. (E) Cell apoptosis‐related proteins levels were detected by Western blotting. (F) The migration of wild‐type cells (WT), single and double KO cells (MYC KO, TAF10 KO, DKO), and Z363‐treated cells were detected by a Transwell migration assay. Scale bar, 20 μm. (G) Representative images showing xenograft tumours at day 28 post‐subcutaneous injection (*n* = 5). (H and I) Tumours were measured and depicted as tumour volume (H) or tumour weight (I). Data shown in A were analysed by two‐way ANOVA. Data shown in C, D, F, H and I were analysed by one‐way ANOVA. Flow cytometry, transwell and blots were representative of three independent experiments. All data are presented as the mean ± SEM of *n* = 3. ***p < .001, **p < .01, *p < .05

Having demonstrated that MYC and TAF10 are coexpressed and epigenetically co‐regulated by TRIP12, we sought to determine whether these genes cooperate functionally to maintain a malignant cell phenotype. First, growth of WT‐type cells and TAF10‐deficient cells was measured after treatment with Z363 at various concentrations. Figure 9A demonstrates that the cytotoxic effect was significantly greater in the absence of TAF10. To confirm the anti‐proliferative effect of double inhibition, MCF7 cells were transfected with CRISPR/Cas9 to generate MYC knockout (MYC KO), TAF10 knockout (TAF10 KO) and MYC/TAF10 double KO (DKO) cell lines; Western blotting confirmed the dependability of the cell lines (Figure 9B). When both TAF10 and MYC were absent from these cells, CCK‐8 assays revealed a synergistic growth inhibitory effect (Figure 9C). In addition, Z363 significantly inhibited cell proliferation (Figure 9C). Hence, Z363 inhibits cell proliferation by co‐regulating TAF10 and MYC.

Using flow cytometry, we observed significant apoptosis in DKO cells (Figure 9D). Z363 was also observed to induce apoptosis (Figure 9D). Regarding apoptosis‐related proteins, double‐KO cells showed complete activation of p53, whereas DKO cells displayed significant alterations (Figure 9E). According to these findings, Z363 co‐regulates TAF10 and MYC to promote apoptosis.

Based on these findings, we evaluated cell migration. Z363 inhibited cell migration by co‐regulating TAF10 and MYC (Figure 9F). MCF7 cells (WT, MYC KO, TAF10 KO and DKO) were injected into the flanks of BALB/c nude mice to determine whether these effects translated to a change in tumour growth. Compared with the WT group, mice treated with Z363 (i.h., 60 mg/kg, twice daily) had a decreased incidence of tumours. Then, tumour development was followed for 4 weeks (Figures 9G–I). At the conclusion of the experiment, the tumours of the DKO group regressed more rapidly than those of the control group. After treatment of Z363, the tumours in the Z363 group began to diminish and were significantly smaller after the experiment. Overall, the DKO group and Z363 group inhibited tumour growth more effectively. These findings suggest that co‐inhibition of MYC and TAF10 synergistically reduces cell proliferation and tumour growth.

## 4 DISCUSSION

MYC is a transcription factor that regulates numerous genes involved in multiple biological processes, such as cell growth, proliferation and apoptosis.^[^ ^20^, ^32^, ^33^ ^]^

Phosphorylation is among the post‐translational modifications that regulate MYC stability.^[^ ^24^, ^25^, ^34^ ^]^ Between Ser62 and Thr58, Ser62 phosphorylation, predominantly by ERK, stabilises MYC.^[^ ^35^, ^36^, ^37^ ^]^ MYC is phosphorylated at Thr58, and then E3 ligase is recruited, leading to MYC protein degradation. MYC, for example, was found to be a proteasomal degradation target of FBXO32.^[^ ^31^ ^]^ Importantly, FBXO32 recognised Thr58‐phosphorylated MYC.

**FIGURE 10:**
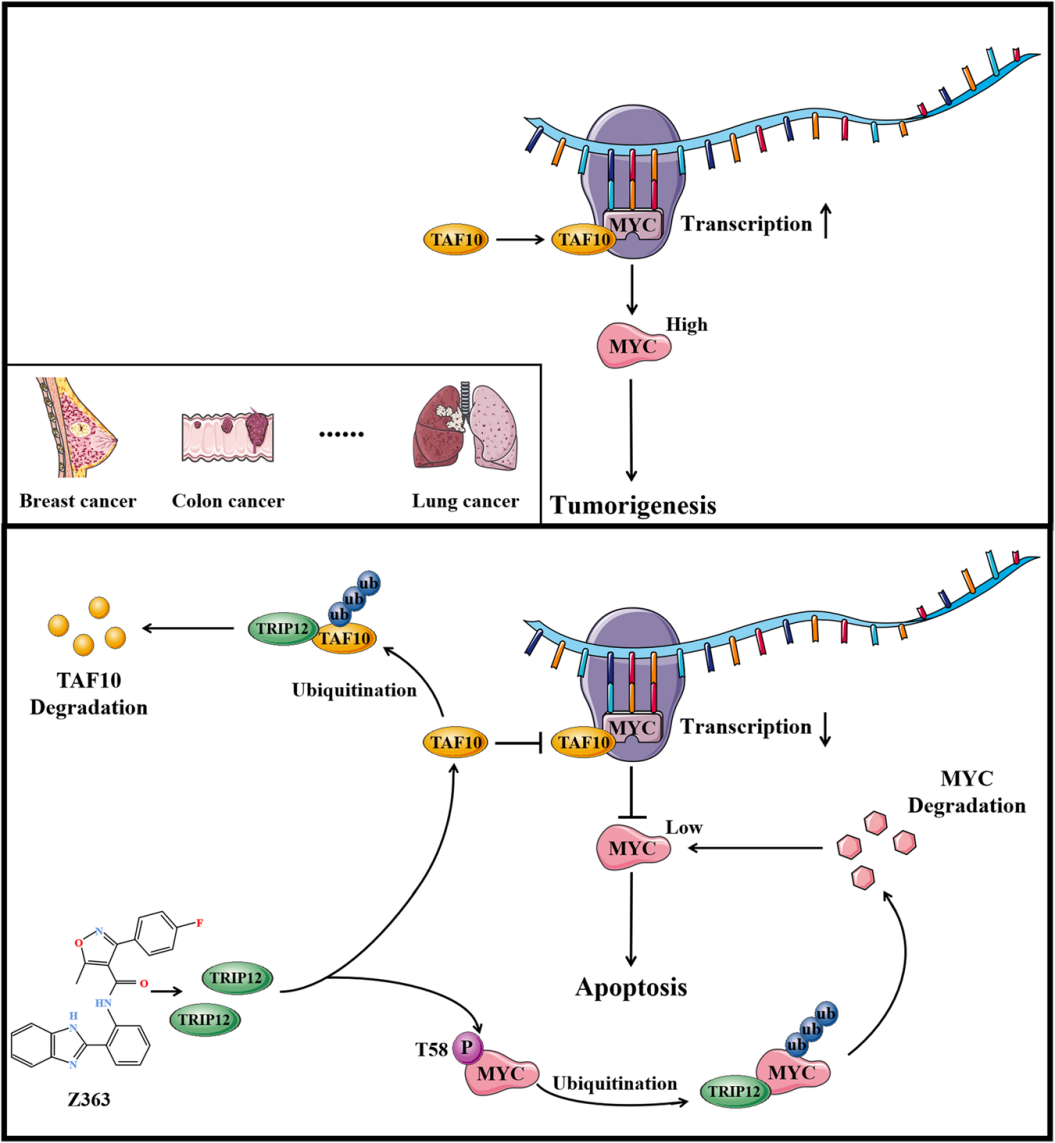
Schematic representation of Z363‐induced anti‐tumour effect in cancer cells

In our study, the small molecule Z363 promoted MYC degradation by increasing MYC phosphorylation on T58, suggesting that Z363‐dependent MYC degradation may involve additional mechanisms (Figure 10). We identified TRIP12 as an E3 ligase of MYC to pursue the molecular mechanism by which Z363 regulates MYC. TRIP12 is a homologous member of the E6‐AP C‐Terminus (HECT) E3 ubiquitin ligase family, which consists of 28 members.^[^ ^38^, ^39^, ^40^, ^41^ ^]^ TRIP12 is essential for Z363‐dependent apoptosis (Figure 5). TRIP12 decreases MYC protein stability by modulating phosphorylation of MYC‐threonine 58. Increased MYC degradation by Thr58 phosphorylation reduces MYC levels, resulting in apoptosis‐inducing cell death. TRIP12 and TAF10 interact to dynamically regulate MYC levels. Consequently, dynamic regulation of MYC transcript and protein levels by TRIP12 activities is essential for apoptosis.

TAF10 stimulates MYC transcription via its 116–206 amino acid (aa) segment. TAF10 overexpression increases MYC transcription, whereas 116–206 aa segment loss decreases MYC transcript levels (Figure 1). In our research, the small molecule Z363 promoted degradation of TAF10, thereby regulating MYC transcription.

Since MYC degradation is required for transcriptional pause release, we hypothesise that TRIP12‐mediated degradation of MYC is responsible for pause release and that loss of total MYC expression is compensated for by TAF10‐mediated MYC transcription. Hence, this dual regulation of TAF10 and TRIP12 for MYC transcription and protein stability is maintained in a dynamic manner, with the small molecule Z363 disrupting this equilibrium.

In addition to regulating MYC levels, TRIP12 regulates the function of TAF10 with regard to MYC, resulting in a coordinated relationship. TRIP12 is a 225‐kDa E3 ubiquitin ligase with a HECT (homologous to the E6‐AP carboxyl terminus) domain.^[^ ^40^, ^41^ ^]^ The TRIP12 HECT domain spans residues 1552–1992, is structured into N‐ and C‐lobes and contains a cysteine at position 1959; substitution of this cysteine with alanine eliminates the ubiquitin ligase activity of TRIP12.^[^ ^42^, ^43^, ^44^, ^45^, ^46^ ^]^ The HECT domain enables TRIP12 to participate in diverse protein degradation and signalling pathways by ensuring the catalytic activity of the ubiquitin ligase.^[^ ^43^, ^47^, ^48^ ^]^ This study demonstrates that the HECT domain of TRIP12 is required for TRIP12 to interact with MYC or TAF10. To prevent formation of tumours, it is likely that these interactions are carefully orchestrated to maintain appropriate MYC levels under both normal and signalled cellular conditions.

Overall, application of the anti‐cancer mechanism of the Z363 cooperative interaction in clinical anti‐cancer drug research is possible. Indeed, enhancement of MYC transcriptional activity by TAF10 signalling promotes cell proliferation in cancer cells. According to our study, co‐targeting of the MYC protein with small molecules or genetic knockdown approaches targeting members of these proteins results in cancer cell apoptosis.

Several MYC inhibitors have been recently developed, including small molecules (BEZ235,^[^ ^49^ ^]^ MYCi361,^[^ ^50^ ^]^ MYCi975^[^ ^21^ ^]^) and monoclonal antibodies.^[^ ^51^ ^]^ We discovered a new anti‐cancer small molecule, Z363, co‐regulating TAF10 and MYC. Co‐inhibition of TAF10 and MYC may thus serve as a novel cancer therapeutic strategy.

## CONFLICT OF INTEREST

The authors declare no conflict of interests.

## Supporting information

Supporting Information

Supporting Information

Supporting Information

Supporting Information
