# Supplementary figures and images for: Small molecule Z363 co‐regulates TAF10 and MYC via the E3 ligase TRIP12 to suppress tumour growth
Source: Clin Transl Med. 2023 Jan 13;13(1):e1153. doi: 10.1002/ctm2.1153 (PMC9839843; doi:10.1002/ctm2.1153)

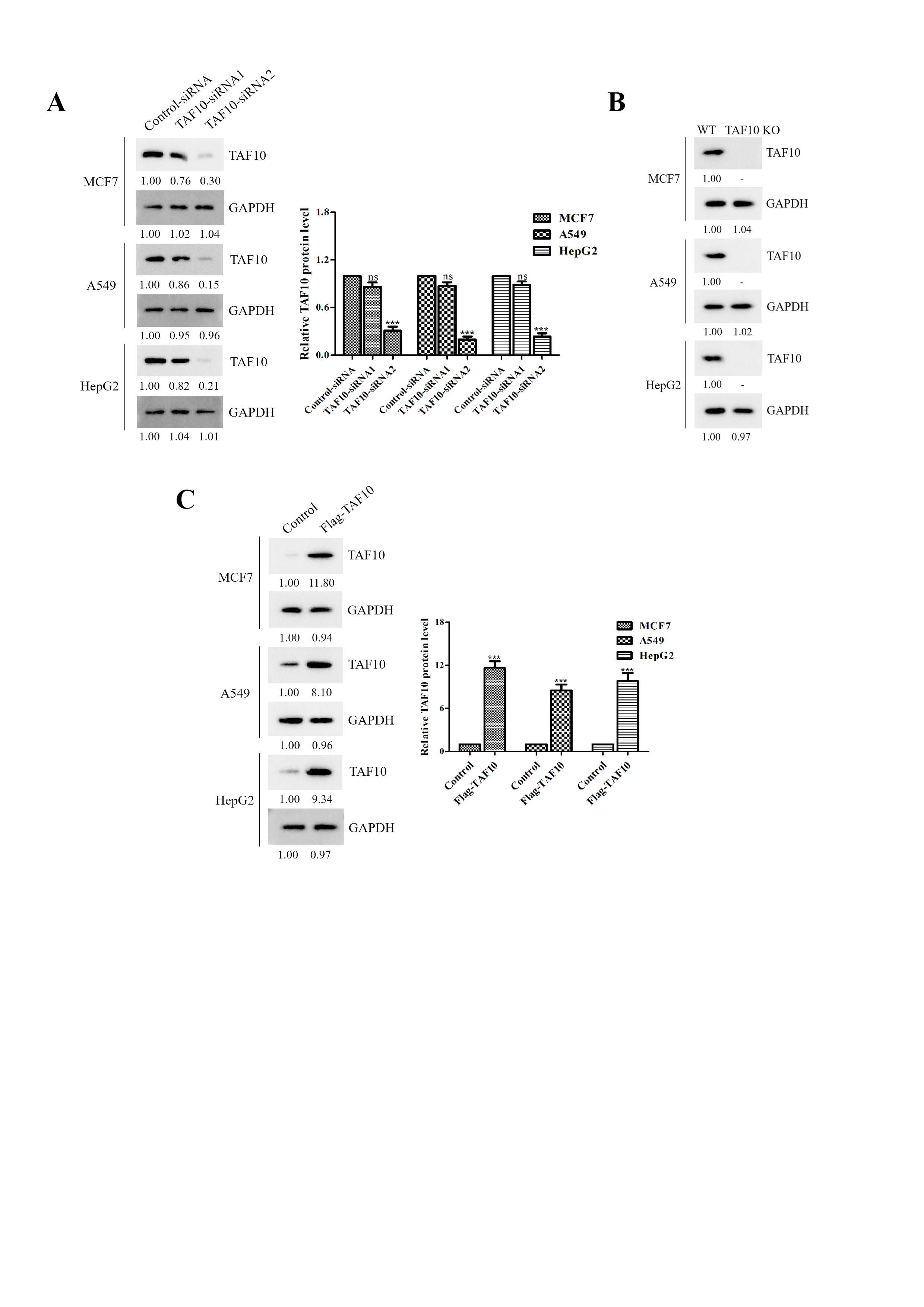

Supplement: Supplementary file 1 — Supporting Information [file CTM2-13-e1153-s004.tif]

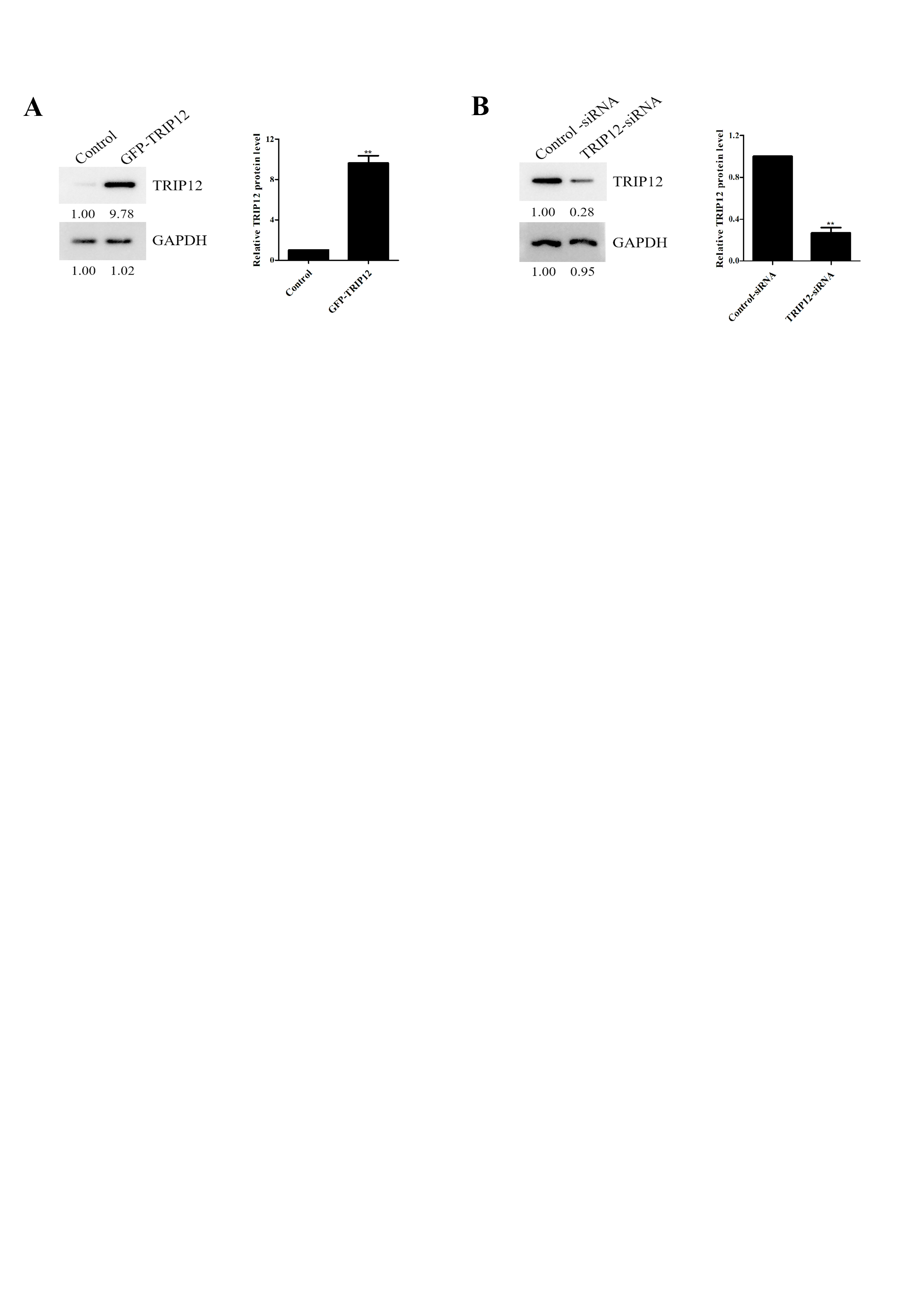

Supplement: Supplementary file 2 — Supporting Information [file CTM2-13-e1153-s003.tif]
