# Supplementary material for: Small molecule Z363 co‐regulates TAF10 and MYC via the E3 ligase TRIP12 to suppress tumour growth
Source: Clin Transl Med. 2023 Jan 13;13(1):e1153. doi: 10.1002/ctm2.1153 (PMC9839843; doi:10.1002/ctm2.1153)
Supplement: Supplementary file 3 — Supporting Information [file CTM2-13-e1153-s001.docx]

Supplementary Table 1

In vitro anticancer activities of small molecules against MCF7 cell lines

| Name | MCF7/IC50(μg/mL)  (n=3) | Solubility | Name | MCF7/IC50(μg/mL)  (n=3) | Solubility |
| --- | --- | --- | --- | --- | --- |
| Z236230776 | 38.6±4.880 | DMSO | Z98332453 | - | DMSO |
| Z254586426 | - | DMSO | Z109826290 | 10.57±2.864 | DMSO |
| Z1244904919 | 47.92±11.585 | DMSO | Z1797830468 | - | DMSO |
| Z25729516 | - | DMSO | Z1754384966 | - | DMSO |
| Z106460362 | 26.21±5.285 | DMSO | Z1601318070 | - | DMSO |
| Z446558384 | - | DMSO | Z363119456 | 4.922±1.784 | DMSO |
| Z1798238306 | - | DMSO | Z73433619 | 4.767±0.9145 | DMSO |
| Z1797022964 | - | DMSO | Z812501892 | - | DMSO |
| Z434402230 | - | DMSO | Z225731922 | - | DMSO |
| Z979255430 | - | DMSO | Z1338140894 | - | DMSO |
| Z1869850523 | - | DMSO | Z243074636 | - | DMSO |
| Z225722720 | - | DMSO | Z85882131 | - | DMSO |
| Z1759959842 | - | DMSO | Z595842248 | - | DMSO |
| Z1175637290 | - | DMSO | Z1303091586 | - | DMSO |
| Z781983374 | - | DMSO | Z1849458643 | - | DMSO |
| Z424991624 | 25.19±4.555 | DMSO | Z1233804881 | - | DMSO |
| Z1139259283 | - | DMSO | Z108564100 | 36.65±8.665 | DMSO |
| Z1134150085 | - | DMSO | Z220618026 | 28.67±1.210 | DMSO |
| Z103867662 | - | DMSO | Z355472054 | - | DMSO |
| Z1068332356 | - | DMSO | Z1657751358 | - | DMSO |
| Z1759961356 | 33.76±6.55 | DMSO | Z1757896319 | - | DMSO |
| Z1148237891 | - | DMSO | Z1455656891 | - | DMSO |
| Z954310956 | - | DMSO | Z1657749856 | - | DMSO |
| Z425512180 | - | DMSO | Z57908343 | - | DMSO |
