# Supplementary material for: Small molecule Z363 co‐regulates TAF10 and MYC via the E3 ligase TRIP12 to suppress tumour growth
Source: Clin Transl Med. 2023 Jan 13;13(1):e1153. doi: 10.1002/ctm2.1153 (PMC9839843; doi:10.1002/ctm2.1153)
Supplement: Supplementary file 4 — Supporting Information [file CTM2-13-e1153-s002.docx]

Supplementary Table 2

| Name | [siRNA](https://www.sciencedirect.com/topics/medicine-and-dentistry/short-hairpin-rna) sequences(5’-3’) |
| --- | --- |
| si-TAF10-1 | 5'-AAUAUCUGAGAUGAAUUUCUG-3’ |
| si-TAF10-2 | 5'-ACAUUGAUGCCAUACUCGCUG-3’ |
| si-TRIP12 | 5'-AGAAUUAGAUUUUCUACUCUC-3' |
| si-SYVN1 | 5'-AACAUUUGAGGAAGAGAAGAA-3’ |
| si-TRIM11 | 5’- CAGAAGUUGUGCCUAUGGA -3’ |
| si-HERC2 | 5′-UGUUUUCAACCAUUUGGAGUC-3′ |
| si-NEDD4L | 5'-GAUUUACCUCCAUAUGAAA-3’ |
| si-SMURF1 | 5'-UGAAGAAGUCUUUCUUUGCAA-3’ |
| si-STUB1 | 5′- AGGCCAAGCACGACAAGUA-3′ |
| si-ITCH | 5′ GCUGUUGUUUGCCAUAGAA55-3′ |
| si-UBR5 | 5′-CAACUUAGAUCUCCUGAAA-3′ |
| si-MDM2 | 5′-AAUAACUUCAAAAGCAAUGGC-3’ |
| si-SMURF2 | 5'-UCGGAAAAAAUCCUUUUUCAC-3’ |
